# Caregiving During the Covid-19 Pandemic Through Publicly-Funded Home- and Community-Based Services

**DOI:** 10.1093/geroni/igaf122.802

**Published:** 2025-12-31

**Authors:** Romil Parikh, Tetyana Shippee, Jack Wolf, Chanee Fabius, Janette Dill, Dana Urbanski, Stephanie Giordano, Eric Jutkowitz

**Affiliations:** University of Minnesota School of Public Health, Minneapolis, Minnesota, United States; University of Minnesota, Minneapolis, Minnesota, United States; University of Minnesota School of Public Health, Minneapolis, Minnesota, United States; Johns Hopkins University, Baltimore, Maryland, United States; University of Minnesota School of Public Health, Minneapolis, Minnesota, United States; Indiana University Bloomington, Bloomington, Indiana, United States; Human Services Research Institute, Cambridge, Massachusetts, United States; University of Minnesota, Minneapolis, Minnesota, United States

## Abstract

Workforce shortages in home- and community-based services (HCBS) were exacerbated during the COVID-19 pandemic. We evaluated changes in HCBS use and consumer-reported unmet HCBS needs for (1) personal care aide (PCA) and (2) caregiver support/respite (CS/R) in 2021-2022 (during the pandemic) versus 2018-2019 (pre-pandemic), in the US National Core Indicators- Aging & Disability Adult Consumer Survey. We included 7143 community-dwelling HCBS consumers (age, ≥65 years, from 11 states). We calculated adjusted odds ratios (aOR) and 95% confidence interval (CI) for HCBS use and consumer-reported unmet HCBS needs for PCA and CS/R using logistic regression, adjusting for sociodemographic and health-related variables (fixed effects), with random intercept for state (to account for clustering by state). For PCA, compared to 2018-2019, during 2021-2022, there was a significant increase in the odds of both PCA use (aOR, 1.24; 95% CI, 1.09, 1.40) and unmet PCA needs (aOR, 1.23; 95% CI, 1.03, 1.46), suggesting that pandemic policy efforts to mitigate service disruptions were insufficient to decrease unmet PCA needs. For CS/R, during 2021-2022, there was a significant increase in the odds of CS/R use (aOR, 1.24; 95% CI, 1.09, 1.40) and a simultaneous significant decrease in the odds of unmet CS/R needs (aOR, 0.49; 95% CI, 0.35, 0.70), suggesting that temporary pandemic policies for CS/R may have successfully abated consumers-reported unmet CS/R service needs. These cross-sectional findings call for more rigorous investigation to identify which temporary pandemic-related CS/R policies were effective; to inform disaster preparedness efforts for mitigating HCBS disruptions during future public health emergencies.

